# Expression of Endogenous Angiotensin-Converting Enzyme 2 in Human Induced Pluripotent Stem Cell-Derived Retinal Organoids

**DOI:** 10.3390/ijms22031320

**Published:** 2021-01-28

**Authors:** Henkie Isahwan Ahmad Mulyadi Lai, Shih-Jie Chou, Yueh Chien, Ping-Hsing Tsai, Chian-Shiu Chien, Chih-Chien Hsu, Ying-Chun Jheng, Mong-Lien Wang, Shih-Hwa Chiou, Yu-Bai Chou, De-Kuang Hwang, Tai-Chi Lin, Shih-Jen Chen, Yi-Ping Yang

**Affiliations:** 1 Institute of Pharmacology, School of Medicine, National Yang-Ming University, Taipei 11217, Taiwan; henkie.lai@gmail.com (H.I.A.M.L.); 49906001@gm.ym.edu.tw (S.-J.C.); figatsai@gmail.com (P.-H.T.); polo661124@yahoo.com.tw (C.-S.C.); shchiou@vghtpe.gov.tw (S.-H.C.); 2 Department of Medical Laboratory, Faculty of Health Sciences, University Selangor, Shah Alam 40000, Selangor, Malaysia; 3 Division of Basic Research, Department of Medical Research, Taipei Veterans General Hospital, Taipei 11217, Taiwan; g39005005@gmail.com (Y.C.); cycom1220@gmail.com (Y.-C.J.); monglien@gmail.com (M.-L.W.); 4 School of Medicine, National Yang-Ming University, Taipei 11217, Taiwan; chihchienym@gmail.com (C.-C.H.); chouchoume@hotmail.com (Y.-B.C.); m95gbk@gmail.com (D.-K.H.); 5 Department of Ophthalmology, Taipei Veterans General Hospital, Taipei 11217, Taiwan; 6 Department of Physical Therapy and Assistive Technology, National Yang-Ming University, Taipei 11217, Taiwan; 7 Institute of Food Safety and Health Risk Assessment, National Yang-Ming University, Taipei 11217, Taiwan

**Keywords:** COVID-19, SARS-CoV-2, SARS-CoV-2 pseudovirus, spike protein, ACE2, organoids, induced pluripotent stem cells

## Abstract

Angiotensin-converting enzyme 2 (ACE2) was identified as the main host cell receptor for the entry of severe acute respiratory syndrome coronavirus 2 (SARS-CoV-2) and its subsequent infection. In some coronavirus disease 2019 (COVID-19) patients, it has been reported that the nervous tissues and the eyes were also affected. However, evidence supporting that the retina is a target tissue for SARS-CoV-2 infection is still lacking. This present study aimed to investigate whether ACE2 expression plays a role in human retinal neurons during SARS-CoV-2 infection. Human induced pluripotent stem cell (hiPSC)-derived retinal organoids and monolayer cultures derived from dissociated retinal organoids were generated. To validate the potential entry of SARS-CoV-2 infection in the retina, we showed that hiPSC-derived retinal organoids and monolayer cultures endogenously express ACE2 and transmembrane serine protease 2 (TMPRSS2) on the mRNA level. Immunofluorescence staining confirmed the protein expression of ACE2 and TMPRSS2 in retinal organoids and monolayer cultures. Furthermore, using the SARS-CoV-2 pseudovirus spike protein with GFP expression system, we found that retinal organoids and monolayer cultures can potentially be infected by the SARS-CoV-2 pseudovirus. Collectively, our findings highlighted the potential of iPSC-derived retinal organoids as the models for ACE2 receptor-based SARS-CoV-2 infection.

## 1. Introduction

Coronaviruses are single-stranded RNA viruses that can infect various animals, resulting in enteric, hepatic, and neurological disorders at various severity levels [1]. In late 2019, the outbreak of coronavirus disease 2019 (COVID-19) largely ravaged the world economy and healthcare system, and scientists have identified severe acute respiratory syndrome coronavirus 2 (SARS-CoV-2) as the causative pathogen of COVID-19. To date, the global COVID-19 cases have surpassed 78 million, and the reported deaths are more than 1.7 million, with rising morbidity and mortality. Coronavirus entry into host cells is predominantly mediated by a spike (S) protein that forms homotrimers protruding from the viral surface [2]. The S protein of SARS-CoV-2 binds to its host receptor, angiotensin-converting enzyme 2 (ACE2), and the viral entry is subsequently primed by the protease of transmembrane serine protease 2 (TMPRSS2), another essential protein required for the fusion of the viral membrane with the host cell membrane [3]. Although the respiratory system is widely accepted as the primary organ for SARS-CoV-2 infection, recent studies have identified cells from other organs that may also be infected by SARS-CoV-2, e.g., blood vessel cells, kidney cells, brain cells, liver cells, and cardiomyocytes [4,5]. For example, the RNA-seq data showed the expression of ACE2 in different cell populations of the kidney organoids, including tubular-like cells and podocytes-like cells [4]. Hao et al. also identified ACE2 expression in oral cavity mucosa, especially in the tongue’s epithelial cells compared to other oral sites [6]. Sang et al. and Jacob et al. demonstrated that the SARS-CoV-2 pseudovirus can successfully infect the nervous system, induced pluripotent stem cell (iPSC)-derived neural cells, and brain organoids [7,8]. Another group reported ACE2 expression in the human cornea and conjunctiva [2]. Furthermore, SARS-CoV-2 was also detected in the postmortem retinal specimens from COVID-19 patients [9]. However, whether the human retina is susceptible to SARS-CoV-2 infection remains unclear.

Induced pluripotent stem cell (iPSC) technologies and patient-derived iPSCs provide opportunities for regenerative medicine, drug discovery, and disease modeling from patient-derived stem cells, such as in cystic fibrosis, Huntington’s, Duchenne muscular dystrophy, Alzheimer, amyotrophic lateral sclerosis, age macular degeneration, and retinitis pigmentosa [10,11,12,13,14,15,16]. Although monolayer cultures are relatively easy to be cultivated, the features that they do not display, the complex tissue organization, limits their bioavailability. In contrast to monolayer cultures, 3D organoids can recapitulate the native histoarchitecture by forming an aggregation of different cell types that allows researchers to investigate the pathology mechanisms in the context of tissue organization microenvironment complexity [17]. Indeed, the applications of iPSC-derived organoids have been extended to basic biological research, medical research, and organ regeneration, such as iPSC-derived lung, brain, vascular, kidney, and retinal organoids [18,19,20,21,22]. Furthermore, human iPSC-derived retinal organoids have been utilized to model human-specific retinal development, disease progression, and drug testing [23]. A series of subsequent studies used human organoids for investigating viral infections such as the ZIKA virus, avian H7N2, and swine H1N1 [24,25]. Moreover, with the rise of global pandemic cases of COVID-19, many studies have used iPSCs as in vitro platforms for investigating the pathogenesis of the SARS-CoV-2 infection or the screening of antiviral drugs against SARS-CoV-2 [26,27,28,29,30].

Given that the SARS-CoV-2 pseudovirus contains the S protein of SARS-CoV-2, it is widely accepted that the entry process of the SARS-CoV-2 pseudovirus highly resembles that of SARS-CoV-2. Using the SARS-CoV-2 pseudovirus for investigating the viral entry process allows experiments to be performed in biosafety level 2 facilities instead of biosafety level 3 [7]. Here, we utilized both human iPSC-derived retinal organoids and retinal monolayer cultures as in vitro platforms to investigate the viral entry of the SARS-CoV-2 pseudovirus and the expression of ACE2 and TMPRSS2. Importantly, our results showed the successful infection of human iPSC-derived retinal organoids and monolayer cultures by the SARS-CoV-2 pseudovirus. To further explore the possible molecular mechanisms of SARS-CoV-2 infectious entry in ACE2 or TMPRSS2-positive retina, we used next-generation sequencing-based bioinformatic approaches and further determined the early cell response after the SARS-CoV-2 pseudovirus infection in retinal organoids. Importantly, our RNA-seq data showed the activation of the defense response, inflammation response, and apoptosis during the SARS-CoV-2 pseudovirus infection in the retinal organoids and monolayer cultures. These findings may highlight that the endogenous expression of ACE2 or TMPRSS2 on retinal organoids and monolayer cultures could serve as the infectious entry and research platforms for SARS-CoV-2 infection.

## 2. Results

### 2.1. Generation of Human Induced Pluripotent Stem Cells (hiPSC) into Retinal Organoids and Monolayer Cultures

**Figure 1:**
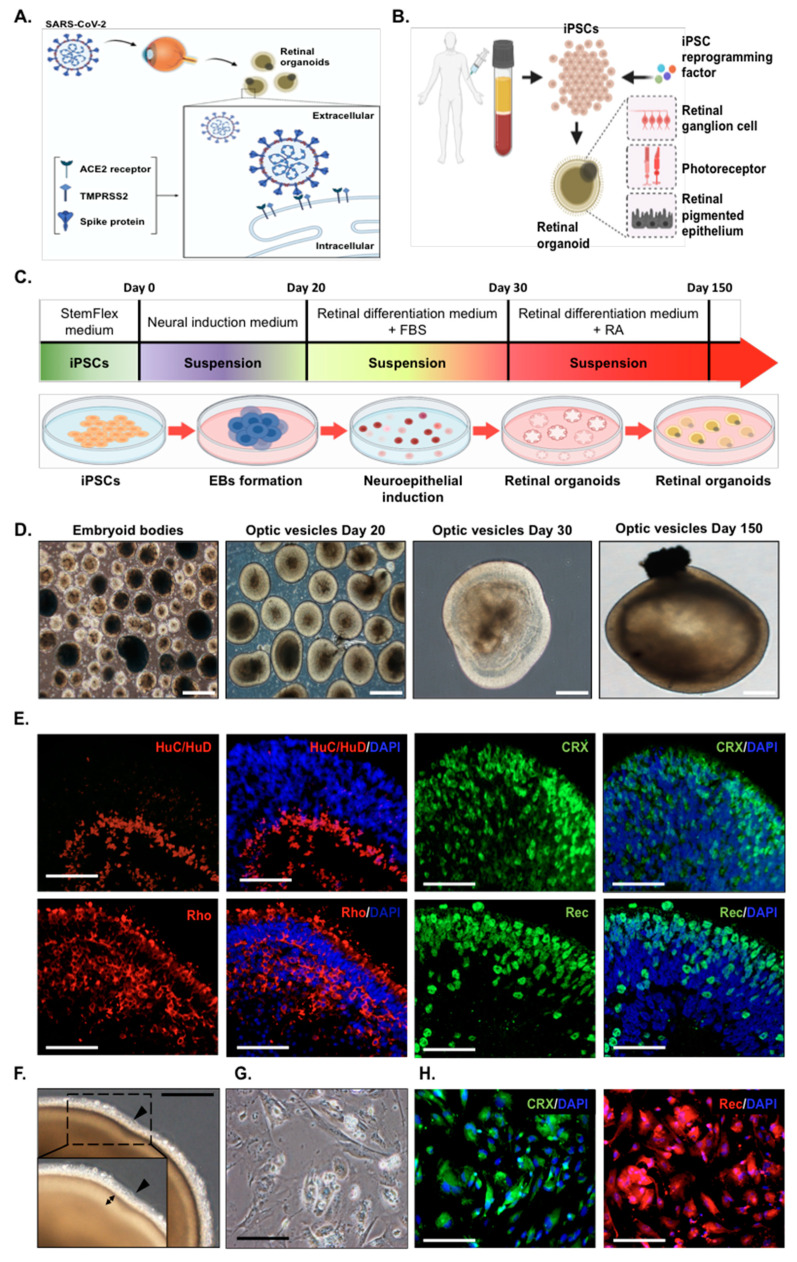
Establishment of human induced pluripotent stem cell (iPSC)-derived retinal organoids and monolayer cultures. (**A**) Schematic showing the examination of severe acute respiratory syndrome coronavirus 2 (SARS-CoV-2) infectivity in the human iPSC-derived retinal organoids. (**B**) Schematic illustrating the generation of human iPSC-derived retinal organoids from human peripheral blood mononuclear cells. (**C**) Schematic diagram illustrating the differentiation protocol for human induced pluripotent stem cell (hiPSC)-derived retinal organoids. (**D**) Bright-field representative images of each stage of the retinal organoid differentiation. Scale bar, 200 µm. (**E**) Immunofluorescence showing the putative retinal markers of day 150 human iPSC-derived organoids. HuC/HuD: neuron marker; cone-rod homeobox (CRX), rhodopsin (Rho), and recoverin [32]: photoreceptor markers. Scale bar, 50 µm. (**F**) Bright-field images showing the representative morphology of day 150 retinal organoids with a dense translucent projection at the apical edge (arrowhead). The double arrow shows the outer nuclear layer. Scale bar, 50 µm. (**G**) Bright-field images of monolayer cultures derived from dissociated retinal organoids. Scale bar, 50 µm. (**H**) Immunofluorescence indicated that the monolayer cultures were positively stained for the CRX (green) and recoverin (red) photoreceptor markers. Scale bar, 50 µm.

Several studies have demonstrated angiotensin-converting enzyme 2 (ACE2) expression in various tissues, including the lung, brain, and kidney. Although the ocular lens was reported as the target of the SARS-CoV-2 infection [31], whether the retina is also an infection target for SARS-CoV-2 remains unclear. To investigate whether the retina expresses endogenous ACE2 and is susceptible to SARS-CoV-2 infection, we first attempted to examine the ACE2 expression on human induced pluripotent stem cell (hiPSC)-derived retinal organoids and monolayer cultures derived from dissociated retinal organoids (Figure 1A). Next, we used the GFP-expressing lentivirus with a SARS-CoV-2 spike (S) protein as the SARS-CoV-2 pseudovirus to mimic the viral entry process of SARS-CoV-2 in host cells. Using such a SARS-CoV-2 pseudovirus system, we sought to evaluate whether the human iPSC-derived 3D retinal organoids system and monolayer cultures derived from dissociated retinal organoids could serve as the potential infectious platform for the viral entry of the SARS-CoV-2 pseudovirus (Figure 1B). We generated human iPSCs from peripheral blood mononuclear cells (PBMCs), and these generated hiPSC colonies were found to exhibit positive alkaline phosphatase activity, embryonic stem cell-like morphology, and a high nucleus-to-cytoplasm ratio (data not shown). These hiPSCs also expressed various pluripotency markers and differentiated into three germ layers in vitro (data not shown).

For the generation of in vitro platforms using hiPSCs, we first applied Ohlemacher’s stepwise methods [33] to differentiate hiPSCs into retinal organoids (Figure 1C). Consistent with previous observations [34,35], embryoid bodies (EBs) were formed by the aggregation of hiPSCs that mimic the process of gastrulation [36]. EBs were further developed into an early eye field state that formed the densely packed pigmentation and was observed in the surrounding cells, which successively resulted in the formation of the optic vesicles (OVs) at day 20 (Figure 1D) [34]. The OVs were further maintained until day 150 and progressively displayed unique morphological features with a bright stratified layer toward the periphery, indicating yielded retinal organoids with a regular layered appearance, and displayed dense translucent projections at the apical edge (Figure 1F). Day 150 retinal organoids showed the expression of photoreceptor-specific markers that were analyzed by immunohistochemistry (Figure 1E). HuC/HuD neurons were observed at the interface between the neuroepithelium, indicating the existence of neuron-like ganglion cells. The emergence of photoreceptor precursors was positive for cone-rod homeobox (CRX) and recoverin, and the rod marker showed the expression of rhodopsin (Rho). Collectively, these data indicated that such a human iPSC-derived retinal organoid system that composes the neural sensory retina and photoreceptor-like neurons might provide an ideal in vitro platform highly similar to retinal tissue [32,37].

In a parallel experiment, we generated a retinal monolayer culture by dissociating the retinal organoids into a single-cell suspension and the subsequent culture on Geltrex precoated culture dishes (Figure 1G). Immunofluorescence staining revealed that the monolayer culture also shared the identical features of putative markers and retained their strong CRX and recoverin expressions (Figure 1H).

### 2.2. Sustained Gene Expression of ACE2 and TMPRSS2 during the Development of Photoreceptor Organoids and Monolayer Cultures

**Figure 2:**
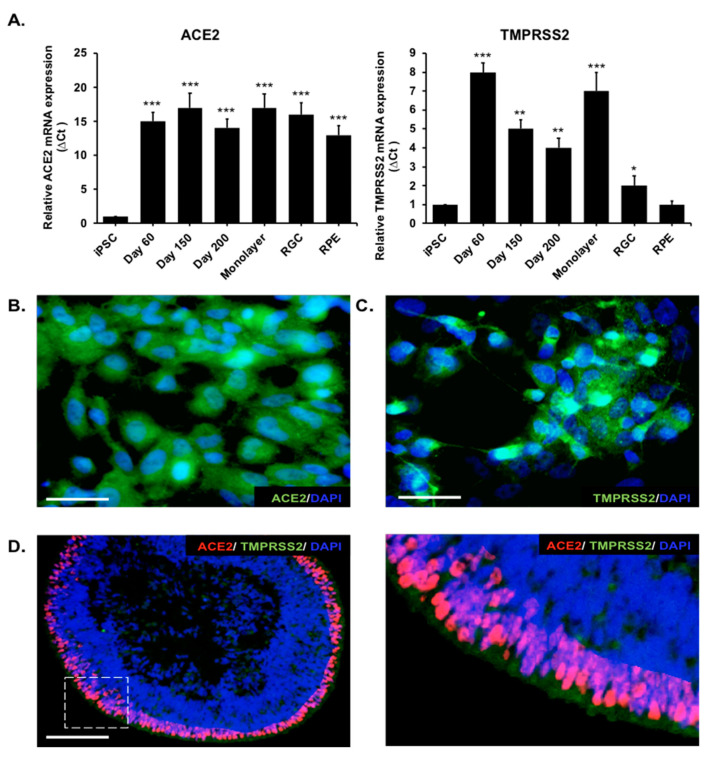
Analysis of angiotensin-converting enzyme 2 (ACE2) and transmembrane serine protease 2 (TMPRSS2) expressions on human iPSC-derived retinal organoids and monolayer cultures. (**A**) qPCR showing the ACE2 and TMPRSS2 gene expressions in retinal organoids (Day 60, Day 150, and Day 200); monolayer cultures; retinal ganglion cells (RGCs); and pigmented epithelium cells (RPEs). The expression of the housekeeping glyceraldehyde-3-phosphate dehydrogenase (GAPDH) gene was used for normalization. * *p* < 0.05, ** *p* < 0.01, and *** *p* < 0.001, and error bars are the standard deviation. (**B**) Immunofluorescence analysis of monolayer cultures with anti-ACE2. ACE2 expression was analyzed in monolayer cultures and stained in green at the cell membrane. Nuclei was stained positive for DAPI (blue). Scale bar, 50 µm. (**C**) The expression of TMPRSS2 (green) in the monolayer cultures. Cell nuclei was stained positive for DAPI. (**D**) The expression and localization of ACE2 and TMPRSS2 in Day-150 retinal organoids were evaluated by an immunofluorescence analysis. The area with ACE2 expression was stained in red and, with TMPRSS2 expression, stained in green. Nuclei were stained positive for DAPI. Right subpanel represents the boxed region of the image in the left subpanel with high magnification.

Angiotensin-converting enzyme 2 (ACE2) can be recognized by both spike proteins of SARS-CoV and SARS-CoV-2 and has been considered the primary receptor for the entry of coronaviruses. TMPRSS2 is an essential protein for priming the SARS-CoV-2 spike protein to fuse with the cell membrane and enable cell entry in the host cells. Therefore, we performed real-time quantitative PCR (qPCR) and immunofluorescence staining to determine the expression of ACE2 and TMPRSS2 in human iPSC-derived retinal organoids and monolayer cultures along the differentiation course (Figure 2). The HiPSCs exhibited a low expression of both ACE2 and TMPRSS2. Similar to that in retinal ganglion cells (RGCs) and retinal pigmented epithelium cells (RPEs), the expression level of ACE2 was increased and sustained from day 60 to day 200 during the whole differentiation period of the retinal organoids (Figure 2A). TMPRSS2 was highly expressed in the retinal organoids on day 60 but slightly decreased by day 200. Remarkably, the monolayer cultures maintained the expression of ACE2 and TMPRSS2, indicating that the dissociation of retinal organoids did not modify the ACE2 and TMPRSS2 expressions. Collectively, the mRNA levels of ACE2 and TMPRSS2 were both expressed in the retinal organoids and monolayer cultures.

We further used an immunofluorescence analysis to examine the ACE2 protein expression and investigated its localization in the retinal monolayer cultures and retinal organoids. Consistent with the findings at the mRNA level, the protein contents of ACE2 and TMPRSS2 were abundant in the monolayer cultures (Figure 2B,C). These results validated the mRNA expression of ACE2 and TMPRSS2 and their protein expressions on the cell membrane of hiPSC-derived monolayer cultures. To examine the expression of ACE2 in the retinal organoids, we performed double-immunofluorescent staining of day-150 retinal organoids (Figure 2D) and found that mature retinal organoids are positively stained for ACE2 and opsin: photoreceptor marker at day 150. Collectively, hiPSC-derived retinal organoids also expressed ACE2 and TMPRSS2 at both the mRNA and protein levels.

### 2.3. Modeling Pseudovirus Infection of SARS-CoV-2 with Retinal Organoids and Monolayer Cultures

**Figure 3:**
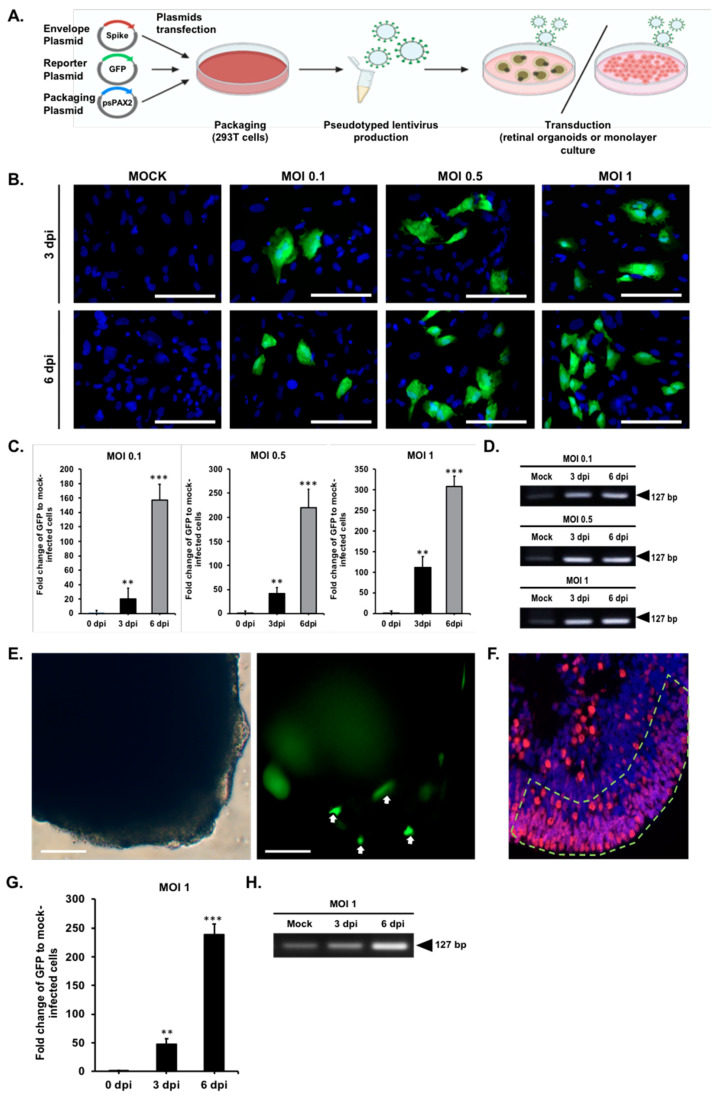
SARS-CoV-2 pseudovirus infection in retinal organoids and monolayer cultures. (**A**) 293T cells were transfected with a plasmid encoding a lentiviral backbone expressing a GFP fluorescent protein, a plasmid-expressing spike protein, and the other necessary proteins essential for virion formation. The transfected cells produced lentiviral particles with a spike protein on their surfaces, and these viral particles were able to infect cells that express the ACE2 receptor. (**B**) Infection of monolayer cultures with the SARS-CoV-2 pseudovirus was performed in uninfected and the SARS-CoV-2 pseudovirus at MOI 0.1, 0.5, and 1 at 3 and 6 days post-infection (dpi). Results represent the mean ± SD from three independent experiments. The presence of a SARS-CoV-2 pseudovirus is shown in green and nuclei in blue. (**C**) The graph shows the fold-change of the SARS-CoV-2 pseudovirus genome copies of uninfected and the SARS-CoV-2 pseudovirus-infected monolayer cultures at the indicated time points: 3 dpi and 6 dpi. Representative data from three independent experiments are shown. ** *p* < 0.01, and *** *p* < 0.001. (**D**) RT-PCR analysis of GFP expression in monolayer cultures infected with different MOI (0.1, 0.5, and 1) and at two time points: 3 dpi and 6 dpi. (**E**) Representative bright-field images of day-150 retinal organoids (left subpanel) infected by the SARS-CoV-2 pseudovirus. The GFP signals (right subpanel) indicate the infection of the SARS-CoV-2 pseudovirus (MOI = 1, dpi = 6). Scale bar, 50 µm. (**F**) Immunofluorescence staining showing ACE2 expression localization at the apical edge of the retinal organoids. (**G,H**) qPCR and RT-PCR analyses of GFP expression in the SARS-CoV-2 pseudovirus-infected retinal organoids at 3 and 6 dpi. ** *p* < 0.01, and *** *p* < 0.001.

To determine the susceptivity of human iPSC-derived retinal organoids and monolayer cultures to SARS-CoV-2 infection, the SARS-CoV-2 pseudovirus was constructed and used to mimic the SARS-CoV-2 viral entry into the host cells (Figure 3A). The SARS-CoV-2 pseudovirus was constructed via transfecting 293T cells with a lentiviral backbone plasmid encoding a fluorescent reporter protein and the SARS-CoV-2 spike (S) protein [38,39]. We subjected retinal organoids (day 150) and monolayer cultures for the SARS-CoV-2 pseudovirus infection to multiplicities of infection of 0.1, 0.5, 1 [40], and uninfected, which was used as the control group (Figure 3B). Since a lentiviral expression vector was used to generate the pseudovirus containing the GFP coding sequence, infection of the cells with the SARS-CoV-2 pseudovirus can be monitored by detecting the GFP signal [41,42]. At three and six days post-infection (dpi), the monolayer cultures were analyzed for viral entry by detecting the GFP signal and counterstained with DAPI to stain the cell nuclei. The observation of the GFP-positive cells indicated that the monolayer cultures were susceptible to the SARS-CoV-2 pseudovirus infection. SARS-CoV-2 pseudovirus genome replication was monitored on days three and six post-infection (dpi) at three different multiplicities of infection [40] (i.e., MOI 0.1, MOI 0.5, and MOI 1). The samples were harvested at three and six dpi after infection and further subjected to qPCR (Figure 3C) and RT-PCR for examining the presence of GFP RNA (Figure 3D). We further analyzed the location of the SARS-CoV-2 pseudovirus containing GFP on the monolayer cultures by double-immunofluorescent staining with anti-recoverin: photoreceptor marker and anti-HuC/HuD: neuron marker in the Supplementary Materials (see Figure S1). Along with the observations that the monolayer cultures expressed ACE2, the infection of the SARS-CoV-2 pseudovirus increased in an MOI-dependent manner.

To further investigate the pathophysiology entry of the SARS-CoV-2 pseudovirus target in retinal eye infections, we employed the hiPSC-derived retinal organoids as the in vitro platform to study their response to the SARS-CoV-2 pseudovirus infection. We treated day-150 retinal organoids with the SARS-CoV-2 pseudovirus at a MOI of one for 24 h (Figure 3E). Since the SARS-CoV-2 pseudovirus contains the coding sequence for GFP, cells infected with the SARS-CoV-2 pseudovirus can be detected with a GFP signal. On post-infection day six, the GFP signals generated by the SARS-CoV-2 pseudovirus were detected on the outer layer of the retinal organoids. Meanwhile, compared with the uninfected control, a mild increase of the cell debris was observed in the culture of infected retinal organoids. qPCR and RT-PCR further showed the time-dependent increase of GFP expression at various time points after infection (0, 3, and 6 dpi; Figure 3F,G). These data demonstrated that human iPSC-derived retinal organoids are susceptible to the SARS-CoV-2 pseudovirus infection, and this confirmed our hypothesis that retinal organoids expressed with ACE2 can be infected with SARS-CoV-2.

### 2.4. Comparative Transcriptome Analysis of the Infected SARS-CoV-2 Pseudovirus

**Figure 4:**
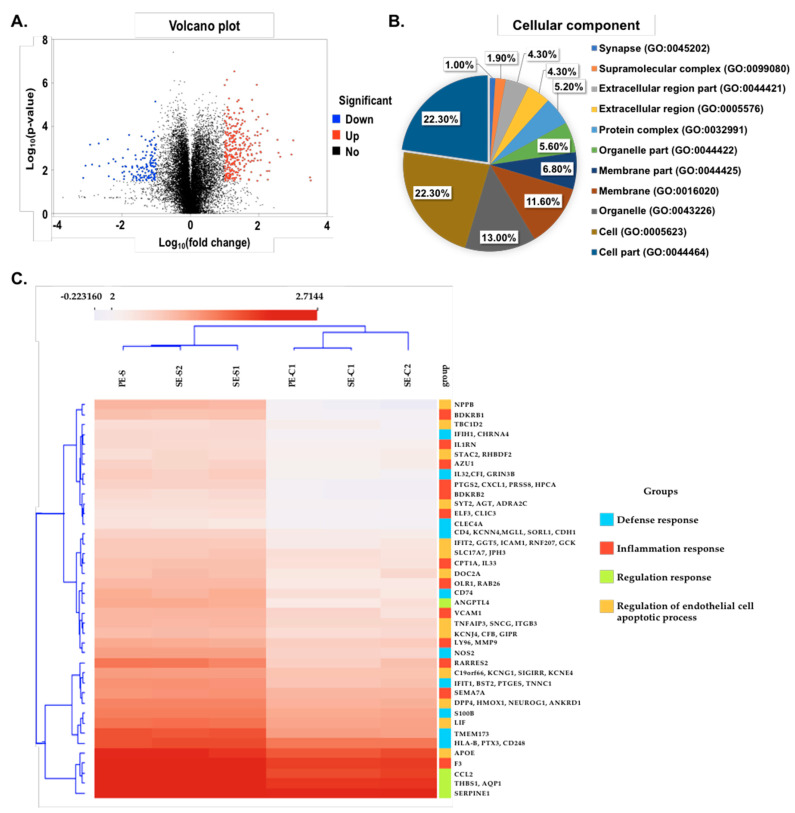
RNA-Seq analysis of hiPSC-derived retinal organoids and monolayer cultures after SARS-CoV-2 pseudovirus infection. (**A**) Volcano plot highlighting the transcript differentially expressed gene during the SARS-CoV-2 pseudovirus infection compared to the parental/uninfected. Red color defines an upregulated expression with a log_2_ (fold change) > 2, and blue defines a downregulated expression with a log_2_ (fold change) < 2. (**B**) Enriched gene oncology (GO) terms of differentially expressed genes in the cells. (**C**) Heatmap expression patterns of parental/uninfected compared to the infected SARS-CoV-2 pseudovirus retinal organoids and monolayer cultures. Red and blue colors indicate differentially expressed genes that were up- (red) and downregulated (blue). PE-S represents the represent the monolayer culture infection of the SARS-CoV-2 pseudovirus, and SE-S1-2 represents the retinal organoid infection of the SARS-CoV-2 pseudovirus. PE-C1 and SE-C1-2 represent the parental/uninfected control for the monolayer cultures and retinal organoids, respectively.

Next-generation sequencing (NGS) has recently been widely used in detecting the infectious strain mutation and biomolecular pathogenesis in the SARS-CoV-2 infection [43,44]. In this study, a transcriptome analysis was further conducted to explore the possible variations of the gene expression patterns in retinal organoids and monolayer cultures before and after the SARS-CoV-2 pseudovirus infection. We collected retinal organoids, infected monolayer cultures, and their corresponding uninfected-treated groups and detected their gene expressions to identify possible differentially expressed genes (Figure 4A). Next, we performed a gene oncology (GO) enrichment analysis to identify the upregulated genes associated with the cellular components (Figure 4B). We categorized both the up- and downregulated genes using Cluster 3.0 software and obtained an overview of the transcriptomic relationships (Figure 4C). Among all the differentially expressed genes in the retinal organoids and monolayer cultures, 10 genes (BDKRB1, BDKRB2, CXCL1, MMP9, F3, RARRES2, PTGS2, IL33, LY96, and VCAM1) were upregulated after the infection by the SARS-CoV-2 pseudovirus. Interestingly, most of these identified genes were closely associated with the inflammation response and endothelial cell apoptosis process. Collectively, the RNA-Seq and bioinformatic analysis revealed that genes related to the inflammation response and apoptosis pathway were upregulated in the SARS-CoV-2 pseudovirus-infected cells, similar to the findings reported in the lung endothelium and vascular tissues of COVID-19 infected people [32,45].

## 3. Discussion

ACE2 is a transmembrane protein expressed on the plasma membrane of the cells in arteries, the heart, lungs, and kidneys. ACE2 was demonstrated to play a significant role in maintaining the homeostasis between blood pressure regulation and angiotensin II expression in the inflammation process [46]. Notably, ACE2 is the receptor responsible for the entry of SARS-CoV-2 into the host cells [47]. The viral spike protein of the SARS-CoV-2 binds to the ACE2 receptors of the host cell and is further cleaved by TMPRSS2 and, thus, increases its viral infectivity [2]. The eye is one of the organs that expresses ACE2, in which ACE expression has been detected on the surface (cornea and conjunctiva) and the inner part of the eye (iris, trabecular meshwork, and retina) [48,49,50]. Consistent with recent studies, our study also demonstrated organoid cultures as a promising approach to model diseases and showed lung and colonic organoid culture effectiveness in being infected by SARS-CoV-2 [51,52]. Organoids for various tissues, intestinal organoids, blood vessel organoids, and brain organoids have been used to better understand the pathological mechanism of SARS-CoV-2 viral tropism [7,47,53]. This study successfully established human iPSC-derived retinal organoids and monolayer cultures as the in vitro model for SARS-CoV-2 pseudovirus infection. The SARS-CoV-2 spike (S) protein gains entry through host ACE2 receptors in host cells, and the serine protease TMPRSS2 assists the priming of the S protein [54]. Although the expression of ACE2 and TMPRSS2 have been well-studied in conjunctival epithelial cells and corneal human iPSC-derived organoids [34], the evidence of RNA and the protein expression of ACE2 and TMPRSS2 in retinal organoids or monolayer cultures are still unclear. To the best of our knowledge, this is the first study investigating ACE2 and TMPRSS2 expression during the differentiation course of retinal organoids from day 60 to day 200 and their dissociated monolayer cultures.

SARS-CoV-2 pseudovirus-based infections have been widely used to study SARS-CoV-2 cellular tropism, recognize the receptors, and assess the viral inhibitors. The present study utilized the SARS-CoV-2 pseudovirus based on the lentiviral system that consists of the S protein on the surface capsid and coded with the GFP sequence. Our results showed that dissociated retinal organoids in monolayer cultures were susceptible to even low MOIs of the SARS-CoV-2 pseudovirus. Additionally, our data showed that the retinal cell infectivity of the SARS-CoV-2 pseudovirus depends on the viral load; a significantly increased GFP was observed in MOI 0.1 to MOI 1. A study conducted on brain neurospheres reported that SARS-CoV-2 shows a significantly increased infection rate in a time-dependent manner [55]. Accordingly, our quantitative analysis of RT-PCR showed that GFP significantly increased during the time course, higher at with six dpi than three dpi. In addition, our findings also supported the ACE2 as an entry portal for the SARS-CoV-2 pseudovirus in retina organoids. According to a recent study using brain organoids, the SARS-CoV-2 antigen was detected in the infected brain organoids after three dpi [8]. Consistent with previous reports on an infection model of the ZIKA virus and SARS-CoV-2, the SARS-CoV-2 pseudovirus induced extensive cell death, as observed by the irregular organoid morphology and cell debris detached from the organoids that can be observed after three dpi as compared to the uninfected control [8,56]. Our results suggested that the retinal organoids and monolayer cultures may serve as a susceptible model for SARS-CoV-2 in vitro infection.

It is particularly interesting that hiPSC-derived retinal organoids and monolayer cultures can be infected by the SARS-CoV-2 pseudovirus. To explore the similarities and differences between the parental cells and SARS-CoV-2-infected host cells, we sought to elucidate the transcriptome of the retinal organoids and monolayer cultures between normal and infected organoids/cells. We used RNA-seq analyses and bioinformatics approaches to identify and characterize the differentially expressed genes in infected organoids and monolayer cultures. A gene ontology enrichment on the 12,602 genes predicted from these transcripts found that they are heavily involved in biological processes, including the regulation of epithelial cell apoptosis process, the inflammatory response, the defense response, the acute inflammatory response, and the regulation of endothelial cell proliferation. Using RNA-seq, we showed that the infection of retinal organoids and monolayer cultures with the SARS-CoV-2 pseudovirus regulated the epithelial cell apoptotic process and inflammatory response. It has been reported that the SARS-CoV infection can act as a signal to trigger apoptosis in the mitochondria [57]. A previous study found that SARS-CoV-2-induced inflammatory responses are the leading cause contributing to the disease progression in COVID-19 patients [58]. Consistent with these findings, our RNA-seq results revealed that inflammatory response genes F3 and SEMA7A are higher in SARS-CoV-2 pseudovirus-infected retinal organoids and monolayer cultures compared to their parental/uninfected controls. Moreover, we observed a significant upregulation of genes associated with the endothelial cell apoptotic process, including CCL2, THBS1, AQP1, and SERPINE1. These data indicated a host cell-specific modulation of cell death by SARS-CoV-2 and an essential role of apoptosis in SARS-CoV-2 pathogenesis [8].

**Figure 5:**
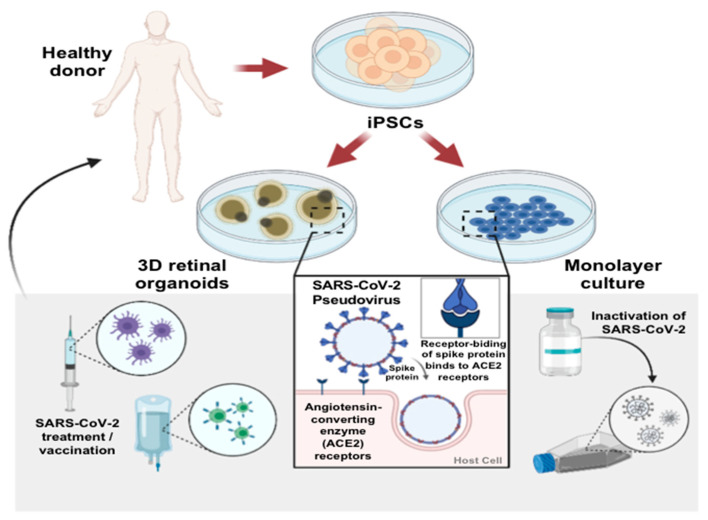
Overview of the SARS-CoV-2 infection in hiPSC-derived retinal organoids and monolayer cultures. General schematic of the experimental workflow of hiPSCs-derived retinal organoids and monolayer cultures. After the in vitro differentiation to the organoids or dissociation into the monolayer cultures, the organoids/cells can be used for disease modeling and therapeutic testing applications.

To date, most SARS-CoV-2 studies have been conducted using immortalized cell lines in monolayers. In conclusion, both retinal organoids and monolayer cultures expressed ACE2 and were susceptible to the SARS-CoV-2 pseudovirus infection. The RNA-seq results indicated that infected retinal organoids and monolayer cultures exhibited cell apoptosis and inflammation. In conclusion, our data demonstrated that human iPSC-derived retinal organoids and monolayer cultures represent excellent disease platforms for the entry portal of the SARS-CoV-2 pathogenesis. These two platforms for the SARS-CoV-2 pseudovirus infection may provide new drug development opportunities and the evaluation of drug efficaciousness and toxicity (Figure 5).

## 4. Materials and Methods

### 4.1. Maintenance and Differentiation of Human-Induced Pluripotent Stem Cells

NTA is a subclone of the human-induced pluripotent stem cell (iPSC) line that was reprogrammed from peripheral blood, were collected following Ethical and Institutional Review Board of Taipei Veterans General Hospital (ID No. 2020-05-004C), using the integration-free Sendai virus carrying the four Yamanaka factors, as described in [22], and their genome integrity and pluripotency were evaluated [22]. NTA was maintained in StemFlex^TM^ Medium (Thermo Scientific, Waltham, MA, USA). NTA was sustained on precoated Geltrex^®^ Matrix (Thermo Scientific, Waltham, MA, USA) culture plates. hiPSCs were passaged every 5 to 6 days at 70% confluency using the nonenzymatic cell dissociation reagent Versene (Thermo Scientific, Waltham, MA, USA)-based protocol.

### 4.2. Differentiation of Retinal Organoids from hiPSCs

The protocol of differentiation of hiPSCs to retinal organoids was adapted with modifications from Ohlemacher et al. [33]. The 70% confluent hiPSCs were detached and dissociated into small clumps with the Versene dissociation protocol and cultured in low attachment culture dishes with 10-µM Y-27632 (Tocris, Minneapolis, MN, USA) to form embryoid bodies (EBs). Neural-induction medium (NIM) consisting of DMEM/F12 (1:1), N2 supplement (Thermo Scientific, Waltham, MA, USA), 1x MEM nonessential amino acids (NEAA), and 2-µg/mL heparin (Millipore Sigma, Burlington, MA, USA) was added at differentiation days (D) 1, D3, and D5 to reach a final ratio 3:1, 1:1, and 1:3 of StemFlex:NIM, respectively. Starting at D7, EBs were cultured in 100% NIM for an additional day. EBs were collected at D7 and plated onto culture plates and cultured in NIM with 10% fetal bovine serum (FBS). Upon the appearance of optic vesicles (OVs; typically, D18–D28) with neuroepithelium morphology, the regions were mechanically removed by scrapers and transferred to a suspension culture in low-attachment 10-cm culture dishes (Corning, New York, NY, USA) in a retinal derived medium (RDM) consisting of DMEM/F12 supplemented with 100-mM taurine (Millipore Sigma, Burlington, MA, USA) and 2-mM GlutaMAX (Thermo Scientific, Waltham, MA, USA). After D60, the RDM was supplemented with 10% fetal bovine serum (FBS; Thermo Scientific, Waltham, MA, USA). Starting at D90, the RDM was supplemented with a N2 supplement and 1-mM retinoid (Millipore Sigma, Burlington, MA, USA) three times a week.

### 4.3. Infection with SARS-CoV-2 Pseudovirus

The SARS-CoV-2 pseudovirus construct was a gift from Dr. Yu-Chi Chou (National RNAi Core from Academia Sinica, Taiwan). The SARS-CoV-2 Pseudovirus used a lentiviral as the backbone, a plasmid-encoding GFP sequence, a plasmid-expressing spike (S) protein as the surface envelope glycoprotein, and the minimal plasmid set of the lentiviral protein necessary to assemble viral particles (Tat, Gal-Pol, and Rev). The CMV promoter was used to drive the expression of GFP. To assay the pseudovirus infection, monolayer cultures were seeded in 6-well pseudovirus plates and were added at the indicated MOIs and centrifuged in the plates at 1200 *g* for 30 min. At 12 h post-infection, cells were washed three times with PBS; then, the infection medium was replaced with fresh medium and kept for 3 to 6 days. At 3 dpi and six dpi, cells were harvested for the qPCR assay or immunofluorescence analysis. For retinal organoids, organoids were seeded in 6-well plates; the pseudovirus was added at the indicated MOIs for 12 h at 37 °C. At 12 h post-infection, the organoids were washed three times with PBS; then, the infection medium was replaced with fresh medium and kept for three days. At three dpi, the organoids were fixed in 4% formaldehyde for 30 min at room temperature for immunohistochemistry. For RNA analysis, cells were lysed in TRIzol.

### 4.4. Immunofluorescence

The 3D organoids and monolayer cultures were then washed twice in PBS and fixed in 4% paraformaldehyde for 15 min. Organoids were permeabilized in 1% Triton X-100, and monolayer cultures were permeabilized in 0.1% Triton X-100; both organoids and retinal neuronal cells were blocked with 5% fetal bovine serum (PBS) for one hour at room temperature. Cells were incubated at room temperature for two hours with primary antibodies (1:100 for anti-recoverin: Sigma-Aldrich AB5585, 1:100 for anti-CRX: Abcam AB140603, 1:100 for anti-HuC/HuD: Thermo Scientific A-21271, 1:100 for anti-rhodopsin: Abcam AB221664, 1:100 for anti-ACE2: GeneTex GTX01160, and 1:100 for anti-TMPRSS: Abcam AB109131). After three washes in PBS, the cells were then incubated with goat anti-rabbit secondary antibody (Alexa Fluor 594-conjugated) or donkey anti-mouse secondary antibody (Alexa Fluor 488-conjugated). The nuclei were counterstained with DAPI (blue) (Abcam). Cells were mounted on slides with a mounting solution.

### 4.5. Quantitative PCR and RT-PCR

Retinal organoids and monolayer cultures were collected and washed twice in PBS, and the total RNA was isolated by TRIzol reagent (Thermo Scientific, Waltham, MA, USA), according to the manufacturer’s protocols. RNA was quantified with NanoDrop 2000 (Thermo Scientific, Waltham, MA, USA); two micrograms of RNA was prepared for the reverse transcription reaction using SuperScript reverse transcription III (Invitrogen) to synthesize complementary DNA strands (cDNA). cDNA was used in the following quantitative PCR (qPCR) and RT-PCR. According to the product’s instructions, the qPCR reaction was performed using the Fast SYBR Green Master Mix. qPCR analysis was performed using three independent biological and technical replicates. The primers were designed using the National Center for Biotechnology Information (NCBI) primer design tool, tested using a standard curve (ACE2_F: GGGATCAGAGATCGGAAGAAGAAA, ACE2_R: AGGAGGTCTGAACATCATCAGTG, TMPRSS2_F: AATCGGTGTGTTCGCCTCTAC, TMPRSS2_R: CGTAGTTCTCGTTCCAGTCGT, GAPDH_F: AGCCACATCGCTCAGACACC, GAPDH_R: GTACTCAGCGGCCAGCATCG). Glyceraldehyde-3-phosphate dehydrogenase (GAPDH) was used as the housekeeping gene, and the relative gene expression of the target genes was determined using the 2^−∆∆Ct^ (delta-delta Ct) method.

### 4.6. RNA-Seq

Total RNA from the infected and uninfected-infected cells was extracted using TRIzol reagent (Invitrogen, CA, USA) and resuspension in RNase-free water. The RNA-seq libraries of polyadenylated RNA were prepared using the TruSeq RNA Library Prep Kit V2 (Illumina, CA, USA), according to the manufacturer’s instructions. RNA-seq libraries for total ribosomal RNA-depleted RNA were prepared using the TruSeq Stranded Total RNA Library Prep Gold (Illumina, CA, USA), according to the manufacturer’s instructions. cDNA libraries were sequenced using an Illumina NextSeq 500 platform.

### 4.7. Bioinformatic Analyses

To cope with the wide range of transcripts, raw reads were aligned to the human genome using the RNA-Seq Alignment Application on Basespace (Illumina, CA, USA), following differential expression analysis using DESeq. Differentially expressed genes (DEGs) were characterized for each sample (*p-*adjusted value <0.05) and were used as a query to search for enriched biological processes and cellular components (gene ontology) and the network analysis of protein interactions using STRING. Heatmaps of the gene expression levels were constructed using the heatmap.2 function from the plot R package. Sparse principal component analysis (sPCA) was performed on the Log_2_ (fold change) values using SPC from the PMA package in R. Volcano plots were constructed using a custom script in R.

### 4.8. Statistical Analysis

Gene expression levels were calculated with relative expression levels (∆Ct). Data were expressed as mean ± standard deviation [58] Statistically significant differences between two groups or among multiple groups were detected by a paired Student’s two-tailed *t*-test and one-way ANOVA with Tukey’s post hoc, respectively, using SPSS Edition 25 software (Chicago, IL, USA). The criterion for significance was set as *p* < 0.05, and highly significant differences in the statistics were accepted if *p* < 0.001. All data presented are representative of at least three independent experiments.

## Data Availability

The data presented in this study are available in this article and supplementary material.

## Supplementary Materials

The following are available online at https://www.mdpi.com/1422-0067/22/3/1320/s1. Figure S1. Representative immunofluorescence staining of infection of monolayer cultures with SARS-CoV-2 pseudovirus.

## Abbreviations

dpi	day post-infection
MOI	multiplicity of infection
COVID-19	Coronavirus disease 2019
TMPRSS2	Transmembrane protease serine 2
ACE2	Angiotensin-converting enzyme 2
qPCR	qualitative polymerase chain reaction
SARS-CoV-2	Severe acute respiratory syndrome-coronavirus-2
iPSC	induced pluripotent stem cell
GFP	green fluorescent protein
