# Supplementary material for: Expression of Endogenous Angiotensin-Converting Enzyme 2 in Human Induced Pluripotent Stem Cell-Derived Retinal Organoids
Source: Int J Mol Sci. 2021 Jan 28;22(3):1320. doi: 10.3390/ijms22031320 (PMC7865454; doi:10.3390/ijms22031320)
Supplement: Supplementary file 1 [file ijms-22-01320-s001.pdf]

SUPPLEMENTARY MATERIAL

Expression of Endogenous Angiotensin-Converting Enzyme 2 in Human Induced Pluripotent Stem Cell-Derived Retinal Organoids

Henkie Isahwan Ahmad Mulyadi Lai [1,2], Shih-Jie Chou [1,3], Yueh Chien [3,4], Ping-Hsing Tsai [1,3], Chian-Shiu Chien [1,3], Chih-Chien Hsu [4,5], Ying-Chun Jheng [3,6], Mong-Lien Wang [3,4,7], Shih-Hwa Chiou [1,3,4,5], Yu-Bai Chou [4,5], Der-Kaung Huang [4,5], Tai-Chi Lin [4,5*], Shih-Jen Chen [4,5*] and Yi-Ping Yang [3,4,7*]

1 Institute of Pharmacology, School of Medicine, National Yang-Ming University, Taipei 11217, Taiwan; henkie@gm.ym.edu.tw (H.I.A.M.L.); 49906001@gm.ym.edu.tw (S.-J.C.); figatsai@gmail.com (P.-H.T.); shchiou@vghtpe.gov.tw (S.-H.C.)
2 Department of Medical Laboratory, Faculty of Health Sciences, University Selangor, Selangor 40000, Malaysia; henkie@unisel.edu.my (H.I.A.M.L.)
3 Division of Basic Research, Department of Medical Research, Taipei Veterans General Hospital, Taipei 11217, Taiwan; g39005005@gmail.com (Y.C.); polo661124@yahoo.com.tw (C.-S. C.); cycom1220@gmail.com (Y.-C.J); monglien@gmail.com (M.-L.W); molly0103@gmail.com (Y.P.Y.)
4 School of Medicine, National Yang-Ming University, Taipei 11217, Taiwan; g39005005@gmail.com (Y.C.); chihchienym@gmail.com (C.-C.H.); chouchoume@hotmail.com (Y.-B.C.); m95gbk@gmail.com (D.-K.H); taichilin@hotmail.com (T.-C.L.); sjchen@vghtpe.gov.tw (S.-J.C)
5 Department of Ophthalmology, Taipei Veterans General Hospital, Taipei 11217, Taiwan
6 Department of Physical Therapy and Assistive Technology, National Yang-Ming University, Taipei, Taiwan
7 Institute of Food Safety and Health Risk Assessment, National Yang-Ming University, Taipei 11217, Taiwan
* Correspondence: molly0103@gmail.com; Tel.: +886-2-2875-7394

S1.

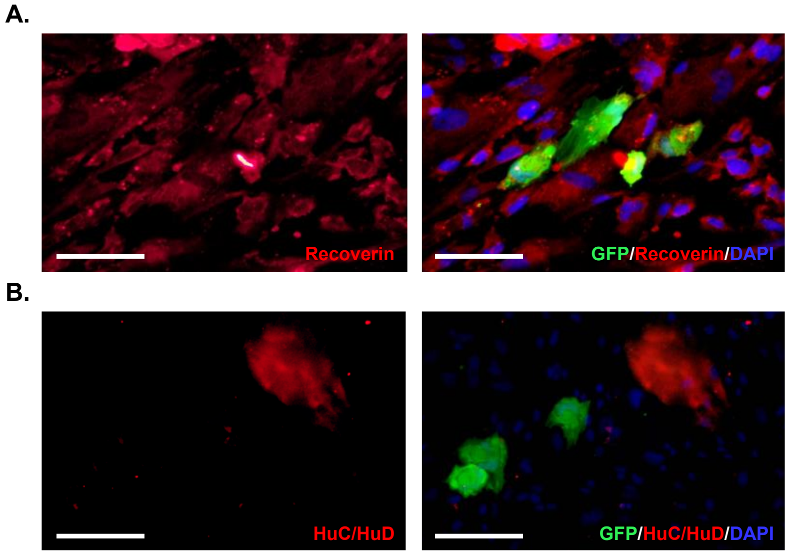

Figure S1. Representative immunofluorescence staining of infection of monolayer cultures with SARS-CoV-2 pseudovirus. (**A**) Double immunofluorescence staining for Recoverin: photoreceptor marker and GFP: SARS-CoV-2 pseudovirus. (**B**) Double immunofluorescence staining for HuC/HuD: neuron marker and GFP: SARS-CoV-2 pseudovirus. The GFP signals (right subpanel) indicated the infection of SARS-CoV-2 pseudovirus (MOI = 1, dpi = 6). Scale bar, 50 μm.
